# The Effects of Extended Release Niacin in Combination with Omega 3 Fatty Acid Supplements in the Treatment of Elevated Lipoprotein (a)

**DOI:** 10.1155/2010/306147

**Published:** 2010-02-24

**Authors:** Alan F. Helmbold, Jennifer N. Slim, Jennifer Morgan, Laudino M. Castillo-Rojas, Eric A. Shry, Ahmad M. Slim

**Affiliations:** Brooke Army Medical Center, MCHE MDC Cardiology Service, 3851 Roger Brooke Drive, Fort Sam Houston, TX 78234-6200, USA

## Abstract

*Objective*. To assess the effectiveness of niacin/fish oil combination therapy in reducing Lipoprotein (a) [Lp(a)] levels after twelve weeks of therapy. *Background*. Lipoprotein (a) accumulates in atherosclerotic lesions and promotes smooth muscle cell growth and is both atherogenic and thrombogenic. A clinical trials of combination therapy for the reduction of Lp(a) has not been previously reported. *Methods*. The study was an observational study following subjects with an elevated Lp(a) (>70 nmol/L) to assess impact of 12 weeks of combination Omega 3FA, niacin, and the Mediterranean diet on Lp(a). *Results*. Twenty three patients were enrolled with 7 patients lost to follow up and 2 patients stopped due to adverse events. The average Lp(a) reduction in the remaining 14 subjects after 12 weeks of combination therapy was 23%  ± 17% [*P* = .003] with a significant association of the reduction of Lp(a) with increasing baseline levels of Lp(a) [R^2^ = 0.633, *P* = .001]. *Conclusions*. There was a significant reduction in Lp(a) levels with combination therapy. A more pronounced effect was noted in patients with higher baseline levels of Lp(a).

## 1. Background/Review of the Literature

According to the Framingham study, a lipoprotein(a) [Lp(a)] >30 mg/dL or >75 nmol/L (assay dependent) is an independent risk factor (RF) correlating to a two- to three-fold increased risk for CAD [1, 2]. When associated with other lipid abnormalities, this risk increases 6–9 times normally [3]. Further, patients with established CAD who have elevated Lp(a) levels are more likely to have acute coronary syndrome (ACS) and in those patients with ACS, baseline Lp(a) levels are predictive of sudden cardiac death [4, 5]. 

The Lp(a) particle has also been shown to be both atherogenic and thrombogenic. Thrombogenicity is partially due to kringle 4 repeats homology to plasminogen thus impairing thrombolysis by competitive inhibition [6, 7]. 

The literature has illustrated reduction of Lp(a) levels via high-dose niacin [8, 9], omega 3FA supplements [10–12], fenofibrate [13], hormone therapy/estrogen replacement therapy [14], tamoxifen therapy [14], aspirin therapy [15], and combination of ascorbic acid 3 grams and lysine 3 grams (Linus Pauling Therapy) [16] as well as L-carnitine [17]. The Lp(a) attachment prevents LDL particle from binding to LDL receptors in the liver and thus statin therapy is ineffective at lowering Lp(a) values [18–20]. There are no prior case series or trials of combination therapy for lowering Lp(a) values; however, a case report from our institution did note a 70% reduction in Lp(a) levels with combination niacin-ER, omega 3FA supplements, and dietary modification [21].

## 2. Research Design and Methods

This was an observational longitudinal study following subjects documented to have an elevated Lp(a) (>75 nmol/L with ELISA method in our institution) to assess impact of 12 weeks of combination of fish oil, niacin, and the Mediterranean diet on Lp(a). Subjects served as their own controls. The appropriate test is a repeated measures ANOVA followed by paired *t*-tests corrected for multiple comparisons. A sample size of 9 subjects was estimated to detect a 70 nmol/L reduction (with a power of 80% and a level of confidence of 95%). We proposed recruiting 20 subjects to allow for possible drop out of 50% in austere military environment. Subjects were candidates for the study if they were 18 years of age or older with an Lp(a) >75 nmol/L. Subjects were excluded if they were pregnant, lactating, diabetic, had a history of gout, recent myocardial infarction (less than 6 weeks), history of liver disease or abnormal liver transaminases (>3x ULN), and could not have taken either omega 3FA supplements or a niacin product during the 3 months proceeding enrollment into the study.

Patients were initially started on extended release niacin (Niaspan) at 500 mg daily and titrated up by increment of 500 mg per week until the target dose of 2 gm was reached. Patients were instructed to eat a small low fat snack and to take enteric coated aspirin 325 mg 30–45 minutes prior to taking niacin to assist with minimizing hot flash symptoms. After titrating niaspan to 2 gm daily, subjects were provided with reading materials related to the Mediterranean diet and encouraged to implement the Mediterranean style of eating as much as possible. Patients were also started on omega 3FA supplements one tab by mouth, three times a day. The fish oil capsules contained 600 mg of EPA and 240 mg of DHA per capsule. After 12 weeks of therapy with 2 gm of extended-release niacin, omega 3FA supplements, and implementation of the Mediterranean diet, final serum concentrations of Lp(a) were obtained. There were no blood draws performed prior to the 12 weeks mark to assess the effect of individual agent on Lp(a). This study does not take into account individual drug effect, but rather the synergistic effect of all the 3 drugs combined. The primary endpoint of the study was the percent change in Lp(a) after 12 weeks of therapy compared to baseline.

## 3. Results

**Figure 1:**
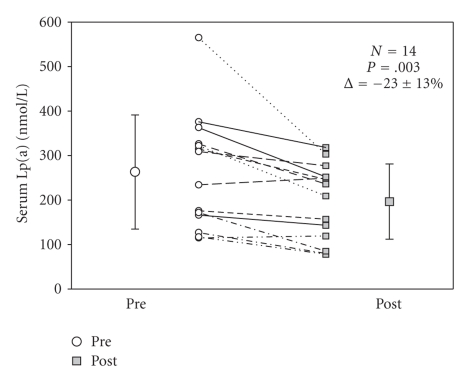
The percent reduction in Lp(a) as observed after 12 weeks of niacin/fish oil combination therapy.

**Figure 2:**
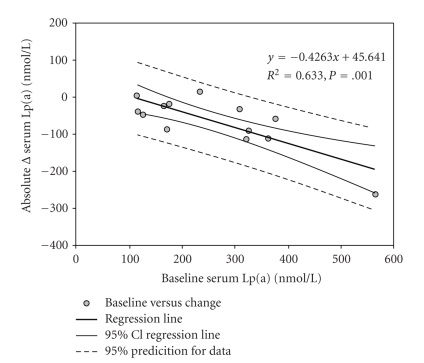
The more pronounced trend in percent reduction in Lp(a) as observed after 12 weeks of niacin/fish oil combination therapy in patients with baseline Lp(a) > 200 nmol/L.

Twenty three subjects were enrolled initially with 7 patients lost to follow up due to deployment and 2 patients stopped due to adverse events. One subject dropped due to severe hot flashes and the other subject due to elevated hepatic transaminases unrelated to the study drug. The total number of subjects who completed the study was 14. The average Lp(a) reduction after 12 weeks of combination therapy was 23 % ± 17% which was statistically significant with a *P* = .003 (see Figure 1). The majority of the reduction was noted in subjects with baseline Lp(a) more than 200 nmol/L which was also statistically significant with a *P* = .001 (see Figure 2). However, the study was not powered to draw conclusions based on subgroup analysis and was merely a pilot observational study aimed at assessing percent reduction with niacin/Fish oil combination therapy.

## 4. Discussion

This study serves as a first step in evaluating the efficacy of combination niacin/fish oil therapy on reducing Lp(a) values. We demonstrated a significant reduction of Lp(a) using a combination regimen of extended-release niacin, omega-3FA, and the Mediterranean diet. Based on our pilot study, there may be linear relationship between the baseline level of Lp(a) and the benefit observed in combination therapy, with baseline Lp(a) levels more than 200 nmol/L deriving the greatest benefit. There are obvious limitations to the study design including small sample size, a significant dropout rate (most due to military deployment during wartime) and short duration of therapy, absence of blood draws prior to the 12 weeks mark to assess the effect of individual agent on Lp(a), as well as not being able to account for individual drug effect on Lp(a). Further, there are some patients who are unable to tolerate this therapy, as evidenced by two patient's withdrawal from the study. Despite these limitations, the study provided us with an insight into the effectiveness of niacin fish oil therapy in treating Lp(a) beyond anecdotal data. It also generates a possible hypothesis that patients with higher baselines Lp(a) (>200 nmol/L) might benefit the most from such therapy. However, such conclusion could be skewed by the marked reduction observed in one subject with baseline Lp(a) exceeding 500 nmol/L and larger studies might be needed to assess if such observed response is clinically significant or just a statistical anomaly. Furthermore, it remains to be investigated if such reduction in Lp(a) with combination therapy translates into reduction in observed cardiovascular outcomes.

## 5. Conclusions

Our pilot study demonstrates that a statistically significant reduction in Lp(a) levels can be achieved with combination therapy. This serves as the basis for future larger prospective, randomized studies to specifically address the reproducibility of our data and the correlation of such reduction with cardiovascular outcome.
